# Exploring attitudes toward physician-assisted death in patients with life-limiting illnesses with varying experiences of palliative care: a pilot study

**DOI:** 10.1186/s12904-018-0304-6

**Published:** 2018-04-04

**Authors:** Patricia Hizo-Abes, Lauren Siegel, Gil Schreier

**Affiliations:** 1 Department of Family Medicine, Schulich School of Medicine & Dentistry, Western University, London, Canada; 2 Lawson Health Research Institute, London Health Sciences Centre, London, Canada; 3 Windsor, Canada

**Keywords:** Palliative care, Medical assistance in dying, Physician-assisted death, Canada

## Abstract

**Background:**

On February 6th, 2015, the Supreme Court of Canada ruled that competent adults suffering intolerably from a grievous and irremediable medical condition have the right to the assistance of a physician in ending their own lives, an act known as physician-assisted death, and later defined as medical assistance in dying, allowing for provision by a physician or a nurse practitioner. As of June 6th, 2016, this is no longer illegal across Canada. There is strong support amongst the general population for physician-assisted death, however there is no recent data on the attitudes of terminally ill patients. Our main objective was to gain information on terminally ill patients’ general and personal attitudes toward physician-assisted death.

**Methods:**

This is an exploratory pilot study. We surveyed three groups of patients with life-limiting diagnoses: one with new referrals to palliative care; one with no palliative care involvement; and one with prior and ongoing management by a palliative care team. Respondents were surveyed twice, approximately two weeks apart, and rated their general attitudes toward physician-assisted death and the hypothetical consideration of physician-assisted death for oneself on a five-point Likert scale at baseline and follow-up. Respondents with new referrals to palliative care were surveyed before and after palliative care consultation. This study was approved by The Western University Health Sciences Research Ethics Board and Lawson Health Research Institute.

**Results:**

We surveyed 102 participants, 70 of whom completed both surveys (31% dropout rate). Participants in all groups predominantly responded between somewhat agree (4 on a 5-point Likert scale) and strongly agree (5 on the Likert scale) when asked about their general attitude toward physician-assisted death. Patients with prior palliative care involvement reported the highest average ratings of hypothetical consideration of physician-assisted death for oneself on a 5-point Likert scale (3.4 at baseline; 3.9 at follow-up), followed by patients with a new palliative consultation (3.2 at baseline; 3.3 at follow-up), and patients with no palliative involvement (2.6 at baseline; 2.9 at follow-up).

**Conclusions:**

Given the preliminary results of this pilot study, we can conclude that terminally ill patients generally agree that physician-assisted death should be available to patients with life-limiting illnesses. Furthermore, descriptive data show a trend for higher hypothetical consideration of physician-assisted death in those patients with prior and ongoing palliative care involvement than patients without palliative involvement. Responses in all groups remained fairly consistent over the two-week period.

**Electronic supplementary material:**

The online version of this article (10.1186/s12904-018-0304-6) contains supplementary material, which is available to authorized users.

## Background

Physician-assisted death (PAD) is an act in which a medical doctor knowingly assists a patient in intentionally ending their own life in order to alleviate suffering [1]. In February 2015, the Supreme Court of Canada ruled that “competent adults…suffering intolerably as a result of a grievous and irremediable medical condition” have the right to PAD. The decision was unanimous and followed multiple challenges since the initial ban in 1972 [2–5]. It was ruled that the existing law infringed upon an individual’s right to autonomy [3, 4, 6]. Bill C-14 entitled “Medical Assistance in Dying” (MAID), was introduced on April 14, 2016 in the Canadian legislature and became law on June 17, 2016 [7]. (For clarity, PAD and MAID refer to the same act, however the term MAID includes provision by either a physician or nurse practitioner and was widely adopted in the later phase of the legislative process, after our study materials had been approved and distributed. Both terms are used throughout the manuscript, PAD in discussing this particular study, and MAID in reference to it in society). Voluntary and informed requests for MAID are considered from mentally competent adults 18 years or older with serious incurable disease, illness, or disability, in an advanced state of irreversible decline toward end of life with foreseeable death. The process involves a waiting period of at least 10 days, and evaluation by two independent physicians or nurse practitioners [7]. The majority of Canadian physicians have expressed opposition to the decision [8–10]. One poll revealed 71.5% of physicians supported the Canadian Medical Association’s policy against participating in euthanasia or PAD [8]. Another found only 44.8% favoured legalizing MAID [10]. In contrast, 78–84% of the Canadian general public supports legal access to MAID [11, 12]. With all the interest in the opinions of lawmakers, politicians, health care providers, and the general public, the absence of the opinions of Canadian patients has gone relatively unnoticed. Canadian studies found approximately two-thirds of patients with HIV/AIDS or incurable cancer supported PAD, and that 39.8% of cancer patients would consider requesting it for themselves [13–16]. These data, however, may not accurately represent patients dying in Canada as cancer and AIDS accounted for only 29.9% and 0.1% of deaths in 2011, respectively [17].

The role of palliative care in PAD is also debated. Some believe PAD is a part of the continuum of palliative care, but 56% of palliative care physicians feel that it fundamentally contradicts the palliative care philosophy and see them as mutually exclusive [8, 9, 18]. Some advocate that it is premature to discuss PAD until palliative care is universally accessible [19]. Unfortunately, based on a 2010 Canadian Senate report, an estimated 70% of Canadians still do not have access to palliative care [8, 20]. Evidence suggests that better pain control and symptom management have not been found to consistently reduce desires for hastened death in the past [21–23]. In Oregon, where PAD is legal, decreasing independence, loss of control, and loss of dignity were the most common concerns expressed by patients choosing PAD, and 92.2% of those who choose PAD had hospice services [24].

Given the recent legislative changes, the lack of up-to-date information on the opinions of patients, and the evolving landscape of palliative care, it is important to explore how patients with life-limiting illnesses currently view PAD to maintain a patient-centered approach. Our primary objective was to explore both general and personal attitudes toward PAD in patients with life-limiting illnesses with varying involvement of palliative care. Secondary objectives included determining whether patients are aware that PAD will be legal in Canada; whether patients are comfortable discussing their views on PAD with family, friends, or their health care providers; and which health care professionals patients would want to provide PAD.

## Methods

### Study design

**Fig. 1:**
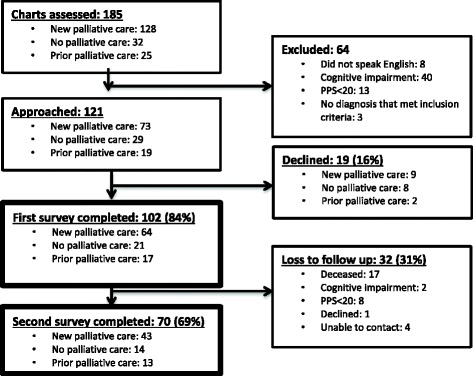
Study flow chart

This was an exploratory pilot study using repeated surveys. We used a convenience sample of adult inpatients and outpatients over age 18 with progressive life-limiting diagnoses, specifically advanced cancer, progressive degenerative conditions, and end organ failure (Additional file 1: Appendix 1). Of the 121 participants that were initially approached, 102 completed the survey resulting in an 84% response rate at baseline. Seventy participants completed the survey at both baseline and follow-up due to a 31% loss to follow-up (Fig. 1).

All surveys were completed at the London Health Sciences Centre in London, Ontario. Recruitment occurred between December 14, 2015 and March 18, 2016. Follow-up encounters and data collection ended April 1, 2016. The study was approved by The Western University Health Sciences Research Ethics Board and Lawson Health Research Institute.

We examined three non-randomized groups. These groups represented terminally ill patients with variable palliative care involvement. The first group did not have palliative care involvement at baseline or follow-up survey administration (No Palliative Care). The second group had initial palliative care consultation between baseline and follow-up survey administration (New Palliative Care). The final group had prior and ongoing palliative care involvement at baseline and follow-up survey administration (Prior Palliative Care).

Potential participants were identified by their most responsible physicians. Each participant was initially approached by a member of his or her circle of care, and then by a study investigator. Those who indicated interest in participating were given the letter of information and a consent form was signed.

### Survey design

A study investigator administered surveys approximately two weeks apart, the first in person, and the second either in person or over the phone. Palliative care consultation took place within 24 h of the first survey in the group with new palliative care involvement. Both surveys included the Edmonton Symptom Assessment System (ESAS) [25] as a validated measure of symptoms, and the Palliative Performance Scale (PPS) [26], as a validated measure of functional status. Both surveys included an explanation of PAD followed by the two questions related to primary outcomes: “Do you think physician-assisted death should be available to patients with serious diseases, illnesses, or disabilities that cannot be cured and who cannot tolerate their suffering?” and “Given that you have a serious disease, illness, or disability, in the future, would you consider physician-assisted death for yourself?” (Additional file 2: Appendix 2). To maximize understanding, the questions were modeled closely after Ontario’s public consultation survey regarding doctor-assisted dying, however surveys were not pilot tested [27]. Respondents rated their level of general support for PAD (1 = strongly disagree to 5 = strongly agree), and their likelihood to consider it for themselves (1 = never consider to 5 = strongly consider) on a five-point Likert scale. With the first survey, we collected demographic data, asked about prior knowledge of PAD, consideration of PAD in the past, and comfort discussing this topic with family, friends, and healthcare professionals. Demographic information and other baseline characteristics were collected on the basis of factors thought to influence desire and attitudes toward PAD [25, 26, 28, 29]. The second survey asked participants which health care providers should provide PAD (Additional file 2: Appendix 2). Survey design and administration was based on the Dillman method [30].

### Statistical analysis

Data analyses for primary and secondary outcomes were limited to descriptive statistics. Missing data were excluded from analyses. Only data from participants that completed both baseline and follow-up surveys were included in the analyses of the primary outcomes to reduce bias. All analyses were performed using SPSS 23.0 for Windows.

## Results

### Baseline characteristics

The baseline characteristics of the three groups were similar overall. Most participants were Caucasian (97%), born in Canada (83%) and had a malignant diagnosis (94%). Eighty percent of participants identified themselves as spiritual and 66% self-reported as religious. Patients with new and prior palliative care were more likely to have a do-not-resuscitate order in place compared to patients without palliative involvement (78%, 53%, and 33%, respectively). The participants’ baseline characteristics are presented in Table 1.

**Table 1:** Baseline characteristics

		No palliative care (*n* = 21)	New palliative care (*n* = 64)	Prior palliative care (*n* = 17)
Age (average ± SD)		68.0 ± 11.2	69.1 ± 12.0	63.8 ± 10.9
Female (n [%])		8 (38)	32 (50)	13 (76)
Inpatients (n [%])		18 (86)	49 (79)	15 (88)
Race	White (n [%])	20 (95)	62 (97)	17 (100)
	Other (n [%])	1 (5)	2 (3)	0 (0)
Born in Canada (n [%])		18 (86)	54 (84)	13 (76)
Marital Status	Single (n [%])	1 (5)	3 (5)	0 (0)
	Married (n [%])	14 (67)	39 (61)	11 (65)
	Divorced (n [%])	3 (14)	10 (16)	3 (18)
	Widowed (n [%])	3 (14)	12 (19)	3 (18)
Education	Post-secondary or higher (n [%])	10 (48)	26 (41)	9 (53)
	High school or lower (n [%])	11 (52)	38 (59)	8 (47)
Spiritual (n [%])		16 (76)	50 (78)	16 (94)
Religious (n [%])		14 (67)	43 (67)	10 (59)
Religion	Catholic (n [%])	9 (43)	22 (34)	6 (35)
	Christian (n [%])	6 (29)	26 (41)	6 (35)
	Other (n [%])	0 (0)	2 (3)	0 (0)
	No religion (n [%])	6 (29)	14 (22)	5 (29)
Diagnosis	Malignant (n [%])	20 (95)	59 (92)	17 (100)
	Non-Malignant (n [%])	1 (5)	5 (8)	0 (0)
Code Status	DNR (do not resuscitate) (n [%])	7 (33)	50 (78)	9 (53)
	Full code (n [%])	10 (48)	13 (20)	5 (29)
	Unsure (n [%])	4 (19)	1 (2)	4 (24)
Year of Diagnosis	Since 2015 (n [%])	12 (57)	39 (61)	10 (59)
	2012–2014 (n [%])	4 (19)	14 (22)	2 (12)
	Before 2012 (n [%])	5 (24)	11 (17)	5 (29)

### Primary outcomes

**Fig. 2:**
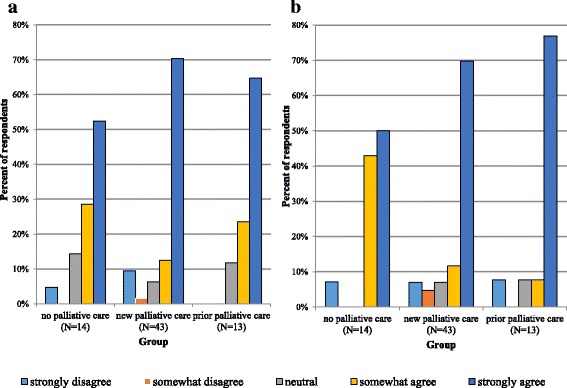
Primary outcome. Responses to question 2 (pretest) “Do you think physician-assisted death should be available to patients with serious diseases, illnesses, or disabilities who cannot be cured and who cannot tolerate their suffering?” on a 5-point Likert scale (1 = strongly disagree, 5 = strongly agree). **a**: Responses by group at baseline. **b**: Responses by group at follow-up

Participants in all groups predominantly responded between somewhat agree (4 on the Likert scale) and strongly agree (5 on the Likert scale) when asked about their general attitude toward PAD. Eighty-one percent at baseline and 93% at follow-up of participants with no palliative care responded either somewhat agree or strongly agree (averaging 4.3 at both time points); 83% at baseline and 82% at follow-up of participants with new palliative consultation responded either somewhat agree or strongly agree (averaging 4.2 and 4.3 respectively); and 89% at baseline and 85% at follow-up of those with prior palliative care responded either somewhat agree or strongly agree (averaging 4.6 and 4.5 respectively) (Fig. 2).

**Fig. 3:**
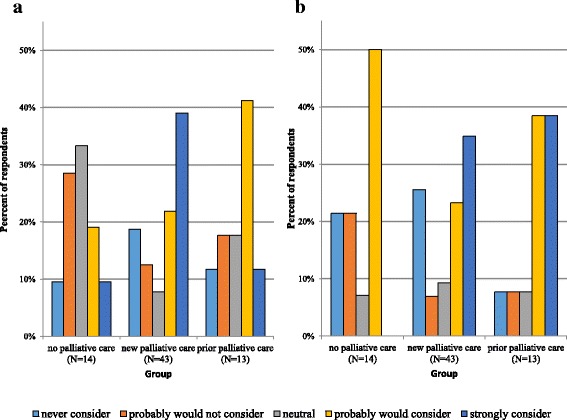
Primary outcome. Responses to question 3b (pretest) “In the future, would you consider physician-assisted death for yourself?” on a 5-point Likert scale (1 = never consider, 5 = strongly consider). **a**: Responses by group at baseline. **b**: Responses by group at follow-up

In terms of a hypothetical consideration of PAD for oneself, responses were more widely distributed across the Likert scale. Participants with no palliative care mostly responded between probably would not consider PAD (2 on the Likert scale) and neutral (3 on the Likert scale), averaging 2.6 at baseline vs. 2.9 at follow-up. Those with new palliative care consultation mainly responded between neutral (3 on the Likert scale) and probably would consider PAD (4 on the Likert scale), averaging 3.2 at baseline vs. 3.3 at follow-up. Those with prior palliative care also mostly responded between neutral (3 on the Likert scale) and probably would consider PAD (4 on the Likert scale), averaging 3.4 at baseline vs. 3.9 at follow-up (Fig. 3).

Slight trends in the demographic data were noticed at baseline. Participants who were born in Canada more somewhat or strongly supported PAD compared to participants born outside of Canada (87% vs. 64% respectively). Participants who were not spiritual more somewhat or strongly supported PAD compared to participants who were spiritual (95% vs. 80% respectively). Participants under 60 years of age more somewhat or strongly supported PAD compared to participants over 60 years (87% vs. 82% respectively). Non-Religious participants more somewhat or strongly supported PAD compared to religious participants (89% vs. 81% respectively).There were no outstanding differences in responses across gender, education, marital status, religion, diagnosis, time since diagnosis, inpatient status, or code status.

Scores on symptom assessment (individual ESAS items), symptom burden (total ESAS score) and functional status (PPS) were relatively stable among all three groups. Pain was higher in patients with new and prior palliative care compared to patients without palliative involvement at both baseline and follow-up (Table 2).

**Table 2:** ESAS and PPS at baseline and follow-up

		No palliative care (*n* = 21)	New palliative care (*n* = 64)	Prior palliative care (*n* = 17)
ESAS at time 1 (average ± SD)	Pain (average ± SD)	1.8 ± 2.8	3.7 ± 3.4	4.3 ± 3.1
	Tiredness (average ± SD)	4.9 ± 2.5	6.1 ± 3.4	6.4 ± 2.9
	Drowsiness (average ± SD)	4.9 ± 2.7	5.2 ± 3.5	5.6 ± 3.6
	Nausea (average ± SD)	0.7 ± 1.8	1.9 ± 2.8	2.4 ± 3.7
	Appetite (average ± SD)	3.0 ± 3.6	4.5 ± 3.9	3.9 ± 3.7
	Dyspnea (average ± SD)	2.9 ± 3.6	2.7 ± 3.3	2.4 ± 2.9
	Depression (average ± SD)	3.0 ± 3.1	3.0 ± 3.5	3.6 ± 3.6
	Anxiety (average ± SD)	2.8 ± 3.0	3.0 ± 3.4	3.3 ± 3.6
	Wellbeing (average ± SD)	3.9 ± 2.7	4.4 ± 2.8	4.8 ± 3.0
	Total distress (average ± SD)	27.9 ± 16.0	34.7 ± 19.4	34.6 ± 21.5
PPS at time 1 (average ± SD)		49.5 ± 9.7	46.7 ± 13.6	44.1 ± 10.0
		No palliative care (*n* = 43)	New palliative care (*n* = 14)	Prior palliative care (*n* = 13)
ESAS at time 2 (average ± SD	Pain (average ± SD)	2.0 ± 2.5	3.7 ± 3.2	3.8 ± 3.0
	Tiredness (average ± SD)	4.7 ± 2.8	6.2 ± 3.0	5.3 ± 3.4
	Drowsiness (average ± SD)	4.5 ± 3.1	4.9 ± 3.6	4.7 ± 3.8
	Nausea (average ± SD)	0.7 ± 1.7	1.9 ± 3.2	1.2 ± 2.1
	Appetite (average ± SD)	2.5 ± 3.4	4.5 ± 4.0	4.3 ± 3.2
	Dyspnea (average ± SD)	2.6 ± 3.4	2.4 ± 3.2	2.2 ± 3.3
	Depression (average ± SD)	2.0 ± 3.5	1.8 ± 3.2	3.8 ± 3.7
	Anxiety (average ± SD)	2.2 ± 3.3	1.8 ± 3.0	3.2 ± 3.8
	Wellbeing (average ± SD)	3.5 ± 3.4	4.1 ± 2.8	5.2 ± 2.5
	Total distress (average ± SD)	26.8 ± 25.3	31.4 ± 18.0	33.7 ± 21.0
PPS at time 2 (average ± SD)		54/3 ± 8.5	47.9 ± 13.0	44.6 ± 11.3

ESAS: Edmonton symptom assessment scale

PPS: Palliative performance scale

### Secondary outcomes

**Fig. 4:**
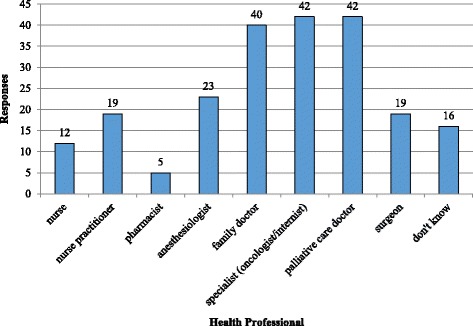
Secondary outcome. Responses to question 3 (post-test) “Who should provide this service?”

Fifty-six percent of participants were aware that PAD would be legal in Canada. One quarter of participants had thought about PAD for themselves in the past. The vast majority of participants felt comfortable discussing their wishes surrounding PAD with their family and their health providers (94% and 92%, respectively). Participants most frequently indicated that family doctors, palliative care doctors, and their respective specialists (e.g. oncologists for cancer patients, cardiologists for heart failure patients, etc.) should provide PAD (Fig. 4). Responses not listed in the figure, but captured under the “other” category included the most responsible physician, groups of physicians, physicians with special training in PAD, or a government-appointed physician. Secondary outcomes are presented in Table 3.

**Table 3:** Secondary outcomes

	No palliative care % respondents (n)	New palliative care % respondents (n)	Prior palliative care % respondents (n)
Did you know that physician-assisted death will be legal in Canada under certain circumstances as of February 6, 2016?			
Yes	38% (8)	59% (38)	65% (11)
No	62% (13)	41% (26)	35% (6)
Given that you have a serious disease, illness, or disability, in the past, have you ever considered physician-assisted death for yourself?			
Yes	5% (1)	30% (19)	29% (5)
No	95% (20)	70% (45)	71% (12)
Would you feel comfortable talking about your wishes with your family or friends?			
Yes	86% (18)	95% (61)	88% (15)
No	14% (3)	5% (3)	12% (2)
Would you feel comfortable discussing your wishes with your healthcare provider?			
Yes	90% (19)	93% (60)	100% (17)
No	10% (2)	6% (4)	0% (0)

## Discussion

The results of this pilot study suggest that patients with terminal illnesses generally support PAD. This finding is consistent with the general public, with 84% of participants expressing support [11, 12]. The responses of most participants remained fairly consistent over the two-week period. Results also suggest that patients with terminal illnesses will hypothetically consider PAD for themselves in the future. Hypothetical consideration of PAD was 53–60%, higher than previously reported in the literature at 39.8% [15]. Prior studies have shown that the desire for hastened death increases over time despite pain control [21, 23]. Our results, although hypothetical, suggest a similar pattern, with the desire for PAD increasing from baseline to follow-up for all three groups. These are important findings given the lack of current information on the attitudes of terminally ill patients towards PAD since the legislative changes in Canada.

A trend in the data was found for participants with new and ongoing palliative involvement to report higher levels of hypothetical consideration of PAD than participants without palliative involvement. This finding is contrary to the belief that palliative care may reduce the desire for hastened death. Possible explanations for this trend may be that patients involved with palliative care are thinking more about death, are more informed about their options, are encouraged to plan ahead, or have a higher symptom burden than non-palliative patients. Previous research suggests that depression and hopelessness are the strongest predictors of desire for hastened death [21]. Although all participants in the current study reported low levels of depression on the ESAS, the prior palliative care group reported slightly higher levels of depression at baseline and follow-up compared to the other two groups of participants, which may also contribute to this trend.

A position statement on PAD released by the American Academy of Hospice and Palliative Medicine (AAHPM) states the ending of suffering by ending life has been held as distinct from palliative care, which relieves suffering without intentionally hastening death [31]. Similarly, a position statement from the Canadian Society of Palliative Care Physicians (CSPCP) found that 80% of members oppose assisted suicide [32]. Our preliminary findings, however, suggest that terminally ill patients may not view PAD and palliative care as mutually exclusive and will hypothetically consider PAD while receiving palliative care. This should not diminish the importance of palliative care and it should not be viewed as a failure of palliative care. Rather, this may indicate a need for a patient-centred approach to integrate PAD into the provision of palliative care in this evolving Canadian landscape of end-of-life care.

The current pilot found that most participants with terminal illnesses are comfortable discussing PAD with their family and health care providers. It is however important to note that although discussions surrounding death and dying are always difficult, there may be a difference in comfort level between patients that are considering PAD and patients that are not considering it. Lastly, most participants feel that family doctors, oncologists, and internists should provide PAD, though a number of other health care professionals would also be acceptable providers. Results showed equal preference for palliative care doctors and specialists such as internists and oncologists to provide this service. This is interesting given that a recent poll of the CSPCP’s members found that 75% of respondents stated that PAD should not be provided by palliative care physicians [9]. This dichotomy of opinions between patient and provider warrants further exploration.

### Strengths and limitations

To our knowledge, this is the first study examining the attitudes of terminally ill patients toward PAD in Canada since the landmark Supreme Court decision in 2015. It is also the first Canadian study examining the impact of palliative care on general and personal attitudes toward PAD. Our study mimics the proposed 10 day waiting period that is required under Canadian legislation by administering surveys approximately 2 weeks apart. Baseline characteristics, including age, race, and religious affiliation as well as validated measures of symptom severity and functional status, were collected to identify trends in demographics [25, 26, 30]. This study provides valuable insight into terminally ill patients’ attitudes towards and opinions of PAD since changes in Canadian legislation, which can be used as a basis for future investigation.

There were a number of limitations to this study. This was a pilot study using convenience sampling with a non-randomized, non-standardized intervention. Initiation of a palliative care consult is highly dependent on subjective assessments by the most responsible physicians. Patients with higher symptom burden or more advanced disease were more likely to be referred to palliative care, which was likely why patients involved with palliative care had higher pain scores at baseline. Palliative care consults and interventions were not standardized, and management varies significantly between providers. Due to the demographics of our centre, this study was done on a largely Caucasian, Christian population with malignancies, limiting the generalizability of findings. Determining prior involvement with palliative care was challenging, as patients may have had varying degrees of palliation by oncologists, internists, and family physicians in the past. For practical reasons, only formal consultation or involvement with specialized palliative care teams was considered as prior involvement with palliative care. Additionally, the new palliative care group may have had as few as one encounter with a palliative care provider during the 10-day period between surveys. This may not be an adequate amount of time to fully realize the impact of palliative care. As the main survey was not validated to ensure sensitivity to change or clarity and flow of questions, all answers should be interpreted as hypothetical desires only. We do not assume or infer any correlation of the hypothetical desires expressed by our participants with actual decisions to pursue or complete MAID. Finally, there was significant loss to follow-up primarily due to progressive morbidity and high rates of mortality in our population of interest.

### Future directions

Further research is needed to examine these findings in a study with a larger sample size to determine statistical significance. Furthermore, a more diverse patient population with chronic and end-stage non-malignant diseases receiving palliative care is needed. Slight trends were noted in the demographic data at baseline for participants who were born in Canada, who were not spiritual, and who were under 60 years of age to be more supportive of PAD in general, and for non-religious participants to be more supportive of PAD for oneself in the future. These findings are preliminary and warrant further investigation. Validation of the survey tool to ensure sensitivity to change is also required. Now that MAID is available in Canada, it would be possible to validate the results of this study and examine whether hypothetical desires or involvement with palliative care correlate with actual requests and completion of MAID. Based on participants’ acceptance of both palliative care and PAD, we suggest that patients do not see a mutual exclusivity between the two services. Further exploration into the patient perspective of PAD and palliative care is needed using qualitative methods. How palliative care and MAID will integrate in Canada is yet to be seen. It is also unclear whether access to palliative care will impact requests for MAID or completion of MAID. The results of this study found preliminary trends in attitudes related to palliative care involvement, suggesting further research is needed to examine these trends in a larger sample of participants. Other aspects of the disease experience that drive attitudes toward PAD should also be considered such as depression and hopelessness. Control, autonomy, independence, dignity, and fear of future suffering have also been shown to influence desires for hastened death and requests for MAID; it is important to explore these challenging issues [22, 24].

## Conclusions

This pilot study suggests that terminally ill patients with and without palliative care equally support the provision of PAD and report hypothetical considerations of PAD that increase over time. A trend emerged that suggests patients with prior and ongoing palliative care and new palliative consultation are more likely to consider PAD for themselves in the future than patients without palliative involvement. These findings serve as a basis for future study.

Despite a widely supported call for a national palliative care strategy, the delivery of palliative care continues to be a challenge across Canada, given the aging population, the increasing burden of chronic diseases, the scarcity of resources, and the evolving expectations of the population [9, 20, 33, 34]. Palliative care specialists cannot be involved with every terminally ill patient, or every request for MAID, but will be pivotal in supporting and empowering other health professionals to engage in complex discussions regarding goals of care, and to help ensure that quality of life, symptom management and end-of-life care are optimized. Our findings suggest that palliative care and MAID may not be viewed as mutually exclusive alternatives to terminally ill patients, suggesting that both must be equally accessible to truly provide patient-centred care.

## Additional files

Additional file 1: **Appendix 1.** Inclusion and exclusion criteria. (DOCX 12 kb)

Additional file 2: **Appendix 2.** All surveys administered to participants. (DOCX 183 kb)

## Abbreviations

MAID: Medical assistance in dying
PAD: Physician-assisted death
