# Supplementary material for: Exploring attitudes toward physician-assisted death in patients with life-limiting illnesses with varying experiences of palliative care: a pilot study
Source: BMC Palliat Care. 2018 Apr 4;17:56. doi: 10.1186/s12904-018-0304-6 (PMC5885418; doi:10.1186/s12904-018-0304-6)
Supplement: Supplementary file 1 — Appendix 1. Inclusion and exclusion criteria. (DOCX 12 kb) [file 12904_2018_304_MOESM1_ESM.docx]

Appendix 1.

|  | **New palliative care** | **Prior palliative care** | **No palliative care** |
| --- | --- | --- | --- |
| Inclusion | 1. Patients diagnosed with at least one of the following advanced life-limiting illnesses:  • Cancer (stage 3 and 4)  • Congestive heart failure (NYHA class 3-4)  • Chronic obstructive pulmonary disease (MRC dyspnea scale grade 3-5)  • End stage renal disease (stage 4-5)  • End stage liver disease (MELD score >30)  • Degenerative neuromuscular conditions  2. Age >18  3. New referral to palliative care team | 1. Patients diagnosed with at least one of the following advanced life-limiting illnesses:  • Cancer (stage 3 and 4)  • Congestive heart failure (NYHA class 3-4)  • Chronic obstructive pulmonary disease (MRC dyspnea scale grade 3-5)  • End stage renal disease (stage 4-5)  • End stage liver disease (MELD score >30)  • Degenerative neuromuscular conditions  2. Age >18  3. Currently followed by palliative care | 1. Patients diagnosed with at least one of the following advanced life-limiting illnesses:  • Cancer (stage 3 and 4)  • Congestive heart failure (NYHA class 3-4)  • Chronic obstructive pulmonary disease (MRC dyspnea scale grade 3-5)  • End stage renal disease (stage 4-5)  • End stage liver disease (MELD score >30)  • Degenerative neuromuscular conditions  2. Age >18 |
| Exclusion | 1. Formal diagnosis of dementia  2. Cognitive impairment with a documented MOCA <26 or MMSE <24  3. Active delirium at either encounter  4. Already involved with a palliative care specialist or team  5. Palliative performance scale score 20% or less at either initial survey or follow up survey  6. Does not speak English  7. Age under 18 years | 1. Formal diagnosis of dementia  2. Cognitive impairment with a documented MOCA <26 or MMSE <24  3. Active delirium at either encounter  4. Already involved with a palliative care specialist or team  5. Palliative performance scale score 20% or less at either initial survey or follow up survey  6. Does not speak English  7. Age under 18 years | 1. Formal diagnosis of dementia  2. Cognitive impairment with a documented MOCA <26 or MMSE <24  3. Active delirium at either encounter  4. Already involved with a palliative care specialist or team  5. Palliative performance scale score 20% or less at either initial survey or follow up survey  6. Does not speak English  7. Age under 18 years  8. New or prior consultation with palliative care service between initial survey and follow-up survey |
