# Supplementary material for: Exploring attitudes toward physician-assisted death in patients with life-limiting illnesses with varying experiences of palliative care: a pilot study
Source: BMC Palliat Care. 2018 Apr 4;17:56. doi: 10.1186/s12904-018-0304-6 (PMC5885418; doi:10.1186/s12904-018-0304-6)
Supplement: Supplementary file 2 — Appendix 2. All surveys administered to participants. (DOCX 183 kb) [file 12904_2018_304_MOESM2_ESM.docx]

Appendix 2.

Survey #_____________

**Background Information**

Age: ____________

Sex: ☐ Male ☐ Female ☐ Other _________________________

Race/ethnicity: _____________________________________

Were you born in Canada? ☐ Yes ☐ No

Marital status:

☐Single

☐Married/Common Law

☐Divorced/Separated

☐Widowed

Highest level of education:

☐Elementary School

☐High School

☐College/University

Are you a spiritual person? ☐ Yes ☐ No

Are you religious? ☐ Yes ☐ No

If so, what is your religion __________________________________________

Diagnosis or medical condition: ______________________________________

Stage: _______________________________

Year of Diagnosis: ____________________________

Are you/have you ever been involved with a palliative care team? ☐ Yes ☐ No

Code status - if you require resuscitation (chest compressions and defibrillation) for example, if your heart stops unexpectedly, what would your wishes be:

☐ Do Not Attempt Resuscitation ☐ Full code ☐ Do not know

Survey #_____________

**Symptom Assessment (ESAS, pretest)**


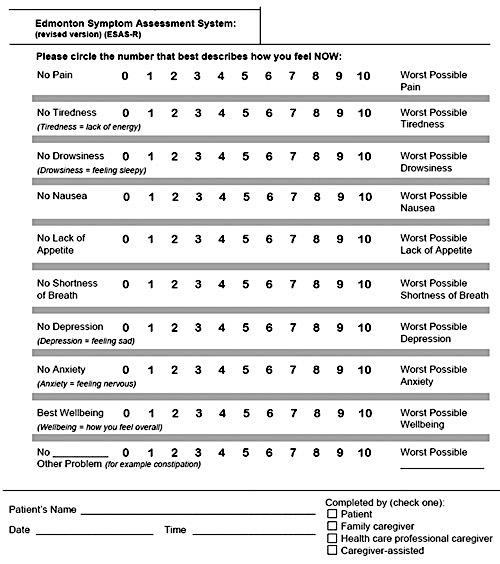


**Functional Assessment (PPS, pretest)**


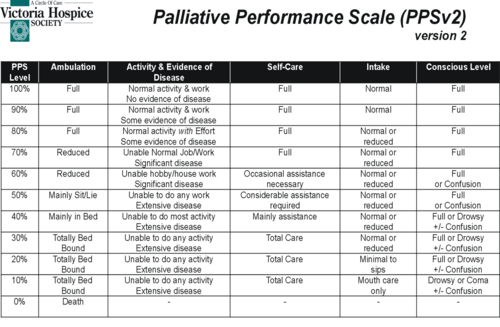


Survey #_____________

**Attitudes toward physician-assisted death in Canada (Pretest)**

Physician-assisted death is when a medical doctor assists a patient in intentionally ending their life in order to alleviate suffering.

**1) Before reading the letter of information, did you know that physician-assisted death will be legal in Canada under certain circumstances as of February 6, 2016?**

☐ Yes ☐ No

**2) Do you think physician-assisted death should be available to patients with serious diseases, illnesses, or disabilities that cannot be cured and who cannot tolerate their suffering?**

Strongly Somewhat Neutral Somewhat Strongly

disagree disagree agree agree

**3) Given that you have a serious disease, illness, or disability:**

**a) In the past, have you ever considered physician-assisted death for yourself?**

☐ Yes ☐ No

**b) In the future, would you consider physician-assisted death for yourself?**

Never Probably would NOT Neutral Probably would Strongly

consider consider consider consider

**4) Would you feel comfortable discussing your wishes with your family and/or friends?**

☐ Yes ☐ No

**5) Would you feel comfortable discussing your wishes with your health care provider?**

☐ Yes ☐ No

**Thank you for your time and participation!**

Survey #_____________

**Symptom Assessment (ESAS, post-test)**


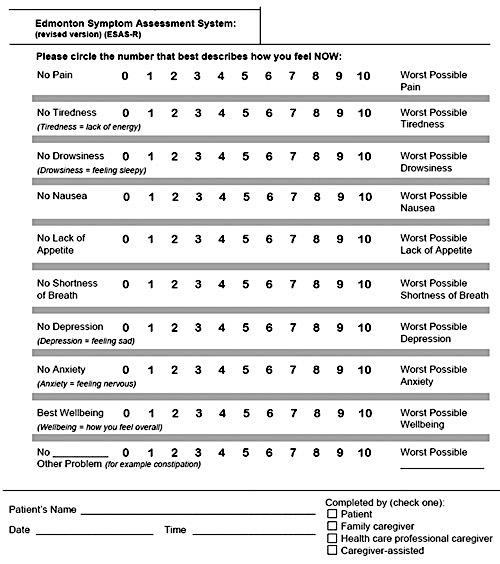


**Functional Assessment (PPS, post-test)**


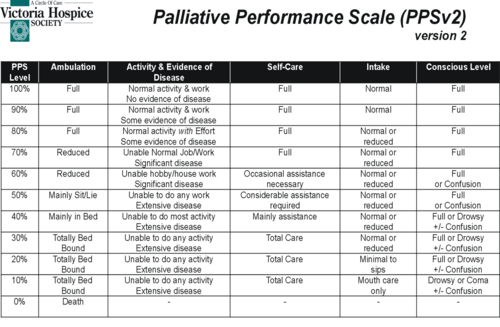


Survey #_____________

**Attitudes toward physician-assisted death in Canada (Post-test)**

Physician-assisted death is when a medical doctor assists a patient in intentionally ending their life in order to alleviate suffering.

**1) Do you think physician-assisted death should be available to patients with serious diseases, illnesses, or disabilities that cannot be cured and who cannot tolerate their suffering?**

Strongly Somewhat Neutral Somewhat Strongly

disagree disagree agree agree

**2) Given that you have a serious disease, illness, or disability, in the future, would you consider physician-assisted death for yourself?**

Never Probably would NOT Neutral Probably would Strongly

consider consider consider consider

**3) Who should provide this service? (select all that apply)**

☐Nurse

☐Nurse Practitioner

☐Pharmacist

☐Anesthesiologist

☐Family doctor

☐Internist or specialist (oncologist/cardiologist/nephrologist/respirologist/hepatologist etc)

☐Palliative care doctor

☐Surgeon

☐Don’t know

☐Other (please specify): _______________________________

**Thank you for your time and participation!**
